# The Glutamatergic System in Primary Somatosensory Neurons and Its Involvement in Sensory Input-Dependent Plasticity

**DOI:** 10.3390/ijms19010069

**Published:** 2017-12-27

**Authors:** Julia Fernández-Montoya, Carlos Avendaño, Pilar Negredo

**Affiliations:** Department of Anatomy, Histology and Neuroscience, Medical School, Autonoma University of Madrid, 28029 Madrid, Spain; julia.fernandez@inv.uam.es (J.F.-M.); carlos.avendano@uam.es (C.A.)

**Keywords:** dorsal root ganglia, trigeminal, glutamate, NMDA, kainate, AMPA, pain, neuropathic

## Abstract

Glutamate is the most common neurotransmitter in both the central and the peripheral nervous system. Glutamate is present in all types of neurons in sensory ganglia, and is released not only from their peripheral and central axon terminals but also from their cell bodies. Consistently, these neurons express ionotropic and metabotropic receptors, as well as other molecules involved in the synthesis, transport and release of the neurotransmitter. Primary sensory neurons are the first neurons in the sensory channels, which receive information from the periphery, and are thus key players in the sensory transduction and in the transmission of this information to higher centers in the pathway. These neurons are tightly enclosed by satellite glial cells, which also express several ionotropic and metabotropic glutamate receptors, and display increases in intracellular calcium accompanying the release of glutamate. One of the main interests in our group has been the study of the implication of the peripheral nervous system in sensory-dependent plasticity. Recently, we have provided novel evidence in favor of morphological changes in first- and second-order neurons of the trigeminal system after sustained alterations of the sensory input. Moreover, these anatomical changes are paralleled by several molecular changes, among which those related to glutamatergic neurotransmission are particularly relevant. In this review, we will describe the state of the art of the glutamatergic system in sensory ganglia and its involvement in input-dependent plasticity, a fundamental ground for advancing our knowledge of the neural mechanisms of learning and adaptation, reaction to injury, and chronic pain.

## 1. Introduction

Glutamate is the most abundant excitatory neurotransmitter in both the central and the peripheral nervous systems, at least in vertebrates. Reasonably, the glutamatergic system has been in the focus of numerous studies that examined how the nervous system adapts to varying conditions of stimulation and activity. While this ability to adapt is the basis for what was the earliest description of “plasticity” applied to the nervous system—“the potential of the brain to adapt to the environment”, according to Cajal [1]—an unequivocal definition of “neural plasticity” is still lacking [2]. No wonder that the term is being applied to a wide variety of events, both structural and functional, from single molecules to complex behaviors. When these events are causally connected, or at least strongly correlated with sensory signals, it is customary to precede the term “plasticity” with qualifiers such as “experience dependent” or “sensory input dependent”. The brain is endowed with multiple mechanisms and capacities to detect and process physicochemical events that reach the sensory receptors, and eventually use that information to generate behavior. When sensory input is altered, brain signals adapt to the new situation, but this “physiological” adaptation may not necessarily be referred to as plasticity. It is when such alteration exceeds certain fuzzy limits—in terms of intensity, quality and/or duration—that the ensuing neural events are generally accepted as revealing plastic capacities of the neural tissue. In a similar vein, developmental events from synapto- or neuritogenesis to brain organogenesis are left outside the scope of the present review, although in many of them the glutamatergic system plays important roles [3,4,5,6].

**Figure 1:**
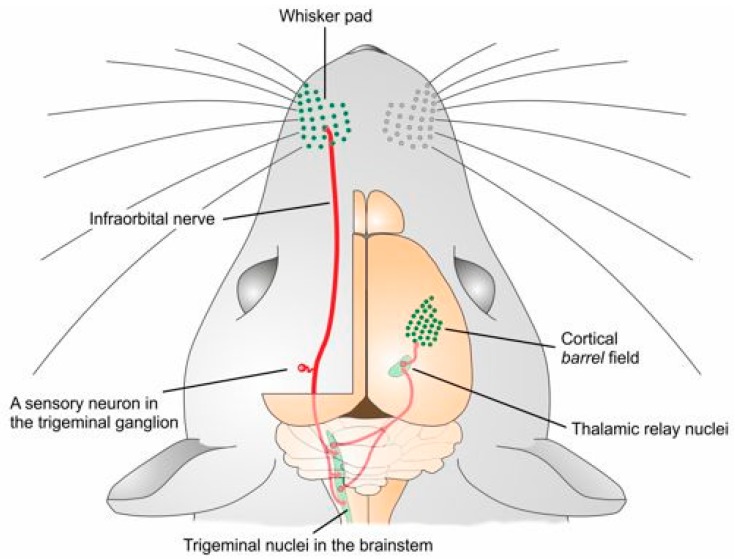
Schematic drawing of the trigeminal somatosensory pathway. Each vibrissal follicle in the whisker pad is innervated by several peripheral endings of primary afferents. The somata of primary sensory neurons are located in the trigeminal ganglion; the central projections of these neurons distribute their terminal arbors in the trigeminal nuclei in the brain stem. These nuclei project densely and in a highly organized spatial pattern to the contralateral thalamus, which, in turn, projects to the somatosensory cortex. Somatosensory information from other bodily regions is collected by primary sensory neurons in the dorsal root ganglion, transferred to the spinal cord and lower brain stem, and eventually projected to the thalamus and the cortex in a comparable manner (not shown).

Sensory information in the somatosensory system arises in sensory receptors and is conveyed and processed along several pathways. A distinctive feature in this system is the precise point-to-point somatotopic pattern adopted by the connections from the receptor sheet through a series of relay stations, up to the somatosensory cortex. Primary sensory neurons support the transduction of the stimulus into a neural code, which is then transferred through their neurites to the central nervous system (CNS). Sensory peripheral afferents consist in the axons of pseudomonopolar neurons whose cell bodies are located in sensory ganglia, such as the dorsal root ganglia (DRG) or the trigeminal ganglia (TG). The single neurite that comes out from the soma bifurcates, with one “peripheral” branch providing sensory innervation to the skin, mucosa and all deep tissues (but the brain and the spinal cord), and another “central” branch leading into the spinal cord or the brainstem (first relay station of the pathway). These first relay neurons (second order neurons) project to the ventral posterior and posterior nuclei of the contralateral thalamus. Finally, thalamic neurons transfer the information to the somatosensory cortex. Somatosensory information from the face, in particular, is channeled to the *barrel* cortex by the trigeminal system (Figure 1).

The trigeminal pathway has been extensively used to study developmental processes, when structural and functional changes are pronounced, and neural plasticity following alterations of sensory input. Once the critical periods of postnatal development are over, sensory cortical areas are still capable of a wide range of structural and functional plasticity, expressed from the molecular level of channels and receptors to the reorganization of circuits and neural networks. The first studies about plasticity in the barrel cortex were made using deprivation paradigms, plucking [7] or trimming [8] some whiskers and leaving others intact. These studies demonstrated the existence of activity-dependent synaptic plasticity in layers IV and II–III of the barrel cortex, in the former diminishing with age and in the latter persisting in adult individuals [9]. In general, this plastic phenomenon after deprivation is characterized by a potentiated response to stimulation of the intact vibrissae and a reduced response to the deprived ones [10].

A large body of data has been gathered on the involvement of the glutamatergic system in these phenomena in the cortex. It was shown in the barrel cortex that deprivation responses are mediated by the induction of Long Term Depression (LTD) in intracortical excitatory synapses established in pyramidal neurons between layers IV and II–III [11]. In fact, since *N*-methyl-d-aspartate receptors (NMDARs) are involved in the development of long term potentiation (LTP), both LTP and LTD have been considered basic blocks of the synaptic plasticity that is behind experience-dependent and pain-associated plasticity in many brain regions [12].

In the mechanisms leading to LTP and LTD multiple glutamate receptors (GluRs) are implicated. LTD is mediated by a slow entry of Ca^2+^ through NMDARs, which in turn activates protein phosphatases that mediate the dephosphorylation of α-amino-3-hydroxy-5-methyl-4-isoxazolepropionic acid receptors (AMPARs) and their internalization [13]. Induction of LTD can also be mediated by metabotropic glutamate receptors (mGluRs) acting conjointly with presynaptic cannabinoid receptors, which, by retrogradely binding endocannabinoids released postsynaptically, lower the probability of neurotransmitters release [14,15]. It is well known that GluRs are also involved in potentiation phenomena, particularly through NMDARs [15], and blocking NMDA receptors prevents the experience-dependent changes in adult barrel cortex [16].

Experience-dependent plasticity in the CNS is strongly associated with changes in the subunit composition of glutamate receptors, as has been extensively documented in the cortex [17,18]. Much less is known about these phenomena in subcortical structures, and the involvement of sensory ganglia in glutamatergic-related plasticity has generally been neglected. Only in disease or injury related plasticity and the pathology of the peripheral nervous system (PNS) have primary sensory neurons become frequently visited subjects of study [19,20,21]. The present review will provide an update of the glutamatergic system as a main general player in sensory experience-dependent plasticity, and will focus particularly on the primary sensory afferents and their ganglia, whose role in such plasticity will surely draw much more attention in the coming years.

## 2. All Primary Sensory Neurons Use Glutamate and Express Glutamate Receptors and Transporters

Primary sensory neuron bodies, as well as their axons, vary widely in size, display an assortment of molecular phenotypes, and likewise show notable differences in functional properties. Neurons responding only or mainly to noxious stimuli, known as nociceptors, are small and their axons are unmyelinated or thinly myelinated. The rest of neurons innervating the skin and other superficial sensory epithelia are larger, sustain myelinated axons, and respond to a variety of mechanical and thermal stimuli with low threshold [22].

Nociceptive as well as non-nociceptive primary sensory neurons express glutamate and release it after stimulation, not only from their central and peripheral terminals [23,24,25], but also from their cell bodies [26]. The first evidence for the presence of glutamate in sensory neurons of the TG and the DRG came from early immunocytochemical studies that showed glutamate-immunoreactivity (IR) in large neurons of the rat DRG and TG but not in autonomic ganglia [27]. Although shortly thereafter glutamate-IR was also shown in small- and medium-sized neurons of rats and other species, only a fraction of neurons was described as glutamate-IR. This fraction varied across studies, ranging from 30% in rat and monkey DRG [28] to 35% in rats, mostly in small neurons [29], up to 65% in cats DRG [30]. Additionally, these neurons express molecules involved in glutamate signaling including proteins involved in its synthesis, transport and release [25,31,32], and others directly related to glutamatergic neurotransmission, such as ionotropic and metabotropic receptors [33,34,35,36,37,38].

### 2.1. Ionotropic Receptors

The terminology for these receptors will follow the NC-IUPHAR recommended nomenclature of ionotropic glutamate receptor subunits [39].

#### 2.1.1. Kainate

Kainate (KA) receptors are homomeric or heteromeric tetramers consisting of different arrangements of five subunits, GluK1, GluK2, GluK3, GluK4 and GluK5 (previously known as GluR5, GluR6, GluR7, KA1 and KA2) that are divided into two groups according to their low and high binding affinity. Homomeric receptors are composed only of low affinity subunits (GluK1, -2, and -3) whereas high affinity subunits (GluK4–5) are present in different combinations in heteromeric receptors [40,41,42].

The presence of presynaptic KA receptors in sensory ganglia has been suggested by reverse transcriptase PCR (rtPCR) in the TG [43], by transmitter release elicited by activation of KA receptors in preparations of dissociated DRGs [44,45], and by immunohistochemistry (IHC) [42,46]. It seems that KA receptors predominate over AMPARs and NMDARs in DRGs, especially in small neurons [46]. In the TG, medium size and small neurons responded to glutamate and KA infusion, and all KA receptor subunits were detected to some extent using rtPCR [43]. After this initial discovery, it was shown that GluK1/GluK5 is the preferential composition of these receptors in DRG and TG neurons [43,47], in contrast with the dominance of the GluK2 subunit in hippocampal and neocortical pyramidal neurons [48,49]. The expression of GluK1/GluK5 receptors shows variations over time, with an increase in the first postnatal week, that has been shown to correspond with the period of greatest developmental plasticity [43].

Low affinity subunits (GluK1, -2, and -3) are expressed presynaptically in primary afferent terminals in the dorsal horn (DH) of the spinal cord, where they are thought to regulate transmitter release [42,45,47,50]. From colocalization studies it has been shown that these subunits are present in both myelinated and unmyelinated primary afferent fibers. Fittingly, the subunits colocalized with tracers applied to the peripheral nerves: the B subunit of cholera toxin CTB (a marker of myelinated axons) in the inner part of lamina II and in lamina III, and with the isolectin of Griffonia simplicifolia IB4 (a marker of unmyelinated axons) in the outer part of lamina II of the DH of the spinal cord [42]. Even though mRNA for GluK2 and GluK3 has been detected in primary afferents, it is unlikely that these subunits alone contribute to form functional receptors. It seems that GluK1 is the critical subunit for activation of the heteromeric complex of presynaptic KA receptors since in its absence (GluK1^−/−^ DRG cells) KA-induced currents are not detected [44,45,47].

The high affinity GluK4 and GluK5 subunits of the KA receptor are expressed in a variety of terminals, including nociceptive afferents, in laminae I–III. These neurons also expressed the GluK1 subunit [51], which is the most abundant in this location [47]. A large majority of GluK1-IR fibers also were positive for the purinergic receptor P2X3 indicating that these fibers belong to the non-peptidergic nociceptive group [51]. GluK1 may form homomeric receptors in some DRG cells or it may combine to form heteromers with the other 4 subunits [41].

The main role of KA receptors at the presynaptic terminal is thought to be the modulation of glutamate release [44,47], since their activation on central endings of DRG neurons may result in an increase or decrease of glutamate release [50]. In addition to their role as ion channels, KA receptors present in DRG neurons can activate a signaling cascade, acting as metabotropic-like receptors. This pathway leads to Ca^2+^ release from intracellular stores and activation of PKC that result in the inhibition of voltage dependent Ca^2+^ channels (VGCC). It has been speculated that this signaling pathway may play a role in KA receptor regulation, helping to maintain the balance of the system [47].

Postsynaptic KA receptors are expressed abundantly throughout the CNS. In the spinal DH, it seems that their localization is predominantly cytoplasmic, suggesting that these receptors are not activated during basal synaptic transmission [21]. In other brain regions KA receptors present differential expression of some of their subunits: GluK5 has a widespread expression in the brain; GluK1 is mainly present in interneurons in the cortex, hippocampus and Purkinje cells in the cerebellum; GluK2 is mostly expressed in hippocampal and cortical pyramidal cells and cerebellar granule cells; GluK3 has the weakest expression, appearing in layer IV of the cortex and dentate gyrus; and GluK4 is found in CA3 pyramidal neurons, dentate gyrus, neocortex and Purkinje cells [52].

#### 2.1.2. AMPA

The ionotropic AMPARs may be composed of different combinations of four subunits, designated GluA1–4 (previously known as GluR1–GluR4) forming tetrameric assemblies of different combinations. Each subunit has a binding site for glutamate, and the receptor is permeable to various cations including Ca^2+^, except when the receptor contains the GluA2 subunit, which renders the channel impermeable to Ca^2+^ [44].

All four types of AMPARs subunits have been shown to exist in sensory ganglia and in their central terminals, exhibiting a differential distribution among neurons of different size. GluA1 seems to be ubiquitously distributed; it is abundant in TG of male rats, as shown by WB [53] and it is present in DRG cells and in both myelinated and unmyelinated fibers, with a preference for small cells [44,46,54], in particular for those expressing substance P (SP) and/or calcitonin gene-related peptide (CGRP), therefore nociceptive peptidergic fibers [55].

Large DRG neurons were immunostained predominantly by anti-GluA2/3; they distributed their central axons in laminae III and IV of DH; and took up the axonal tracer CTB. Hence, they correspond to Aβ and Aδ myelinated fibers that mediate mechanosensitivity and noxious mechanical and thermal stimuli [36,46,54]. On the other hand, small cells were immunostained predominantly by anti-GluA4 or anti-GluA2/4; they colocalized with P2X3 and bound IB4, markers of non-peptidergic and unmyelinated primary nociceptors [36,38]; and their terminals ended in laminae I and II [36,54]. Since GluA4 is expressed in a large number of non-peptidergic primary fibers, which are strongly associated with neuropathic pain, this subunit may be involved in a presynaptic modulation of non-peptidergic nociceptors [38].

Postsynaptic AMPARs have a widespread distribution in the entire brain and are clearly involved in plasticity [56]. Different brain regions are enriched in specific AMPAR subunits. In the DH, all neurons possess the GluA2 subunit; most superficial DH cells also exhibit receptors containing the GluA1 subunit; GluA3 is more weekly expressed in laminae I–II than in deeper laminae; and GluA4 is generally sparse in laminae I–II [21]. It should be noted that AMPARs located in central terminals of primary sensory axons could develop a postsynaptic function too. It seems that they are involved in mechanisms of presynaptic inhibition through a phenomenon known as primary afferent depolarization, traditionally attributed to GABAergic interneurons. In this scenario, endogenously released glutamate can activate presynaptic AMPARs, probably by a spill-over mechanism producing local depolarization at the terminals, depression of action potentials and inhibition of glutamate release from those same terminals [55]. Moreover, AMPAR subunits are transported from the DRG cell bodies to the periphery, as demonstrated by the presence of AMPAR immunoreactivity in the glabrous skin of the rat hindpaw [57,58].

#### 2.1.3. NMDA

NMDARs are heteromeric tetramers composed of three subunits, GluN1, GluN2A–D and GluN3A,B [59]. The presence of the GluN1 is obligatory to form functional receptors. NMDARs subtypes confer different gating and permeation properties to the channel; the presence of GluN2B gives high glutamate sensitivity which is even greater when GluN2C/D are present because of its capacity to reduce the voltage-dependent Mg^2+^ block, a property that is shared by the GluN3 subunit [33].

Expression of GluN1 subunit was shown by WB, real time PCR (RT-PCR) [37], EM and IHC in numerous DRG neurons of all sizes, and also in a large number of fibers, mostly myelinated A-fiber positive for NF200 [37,38,60]. These fibers were found to terminate more abundantly in laminae IIi, III and IV, and among them those labeled with CTB predominated over those binding IB4. No terminal was found to be labeled with CGRP, suggesting that GluN1 is expressed mainly in presumed low-threshold mechanosensitive afferents [60].

RT-PCR experiments detected mRNA expression of GluN2A–D in DRG neurons, showing GluN2B and GluN2D higher expression than GluN2A and GluN2C. Proteins of these subunits were also detected using WB techniques with the exception of GluN2A that was only detected at the mRNA level [37]. IHC and functional data have confirmed that GluN2B and -D predominate in DRG neurons. While the most common NMDAR in A fibers seems to be the diheteromer GluN1/GluN2B [37], C fibers (both peptidergic and non-peptidergic) could also express an additional receptor containing GluN1, GluN2D, and possibly GluN2C subunits [37,44]. GluN1, GluN2A and GluN2B were also detected by WB technique in the TG of adult male rats [53].

Experimental ligation of dorsal roots demonstrated that NMDARs are synthesized in the DRG and transported along the dorsal root towards the central terminals, where they act as presynaptic receptors in primary afferents modulating afferent input [60,61]. Glutamate seems to be involved in sensory transduction too, since expression of GluRs and vesicular transporters was demonstrated in Merkel cells, and GluN1 and GluN2B/A were identified in a zone of the rat sinus hair follicle known to be densely populated by Merkel cells [62].

Postsynaptic NMDARs have a wide distribution in the CNS and are fundamental for normal synaptic function and for synaptic plasticity phenomena [13]. The different subunit expression changes, both during development and in the adult brain, depending on their location. It has been proved that GluN2B and GluN2D are abundant during early development, forming diheteromeric receptors together with GluN1, whereas GluN2A or GluN2C subunits are added progressively during development. While GluN2B is expressed only in the forebrain and GluN2C is enriched in the cerebellum, GluN2A is ubiquitously expressed in the adult brain [63,64], where they can form not only diheteromeric receptors but also triheteromeric, including different GluN2 subunits [65].

#### 2.1.4. Delta

The glutamate ionotropic receptor channels delta1 and delta2, now named GluD1 and GluD2, lack specific ligands, are considered orphan receptors, and their functions remain poorly understood [66,67]. Some recent studies, however, point to a role of GluD2 in the generation of LTD in the cerebellar cortex, which takes place by controlling the phosphorylation and internalization of the GluA2 subunit in Purkinje cell dendrites [68]. While the presence of these receptors in DRG or TG has not been examined, the mRNA coding for GluD1 is strongly expressed in sensory hair cells of the inner ear, as well as in the primary sensory neurons of the cochlear and vestibular ganglia, and the satellite glial cells in the cochlear ganglion [69].

### 2.2. Metabotropic Receptors

The mGluRs form a family of protein G-coupled receptors, which have been classified into three subgroups according to their molecular structure, signaling pathways and pharmacological and physiological properties: Group I receptors (mGluR1a–d and mGluR5a–b) are coupled by Gq proteins to the phospholipase C signal transduction pathway and increase neuronal excitability; Group II (mGluR2 and mGluR3) are coupled to Gi/Go, which inhibit cAMP formation, decreasing neuronal excitability; Group III (mGluR4a–b, mGluR6, mGluR7a–b and mGluR8) are also coupled to Gi/Go, negatively regulate adenylate cyclase activity and decrease excitability [35,70,71,72]. Group I mGluRs are mainly localized at the postsynaptic density, whereas Group II and III are primarily, but not exclusively, located presynaptically, where they serve as auto- and hetero-receptors. In virtue of their synaptic location and their coupling to G-proteins mGluRs can finely tune the cellular response to glutamate signaling: postsynaptically they can modulate neuronal excitability, and presynaptically they can act as auto- and hetero-receptors and modulate neurotransmitter release [70,71].

In primary sensory ganglia mGluRs, first identified by WB and IHC, are expressed by neurons of different sizes in the TG [73]. Shortly thereafter, the presence of mRNA for a number of mGluRs was demonstrated by in situ hybridization in TG and DRG, as well as in numerous regions of the CNS [74]. Further studies showed that all three groups of mGluRs are present in sensory ganglia and in primary sensory afferents.

#### 2.2.1. Group I

Using immunolabeling, the mGluR5 subunit is present in small to medium size DRG neurons of rats and humans, as well as in the DH of the spinal cord [72]. In this study, labeling was markedly heterogeneous, both in intensity, since small diameter DRG neurons were most intensely stained than larger neurons, and in regional distribution, with strong mGluR5-IR confined within the superficial layers of the DH. Using IHC and pharmacological approaches, Walker [75] demonstrated that mGluR5 appears in both the central and the peripheral terminals of primary afferents, and that most neurons expressing this receptor also expressed the vanilloid receptor 1 (VR1), a marker of small nociceptive neurons. The intraplantar injection of a mGluR5 agonist was capable of evoking mechanical hyperalgesia and this effect was prevented by the application of a selective antagonist, suggesting a functional role of mGluR5 in inflammatory hyperalgesia [74]. It is now widely accepted that peripheral Group I mGluRs appear to be strategically positioned to activate nociceptors and modulate sensitization, making them suitable candidates for topical analgesic treatments [71].

Only 7% of DRG cells, most of them small- and medium-sized, express mGluR1, as shown by IHC and stereological techniques [35]. Approximately half of these cells also coexpress mGluR8 and the other half express mGluR2/3. There is evidence that both types of Group I receptors are also present in the peripheral branches of DRG neurons. For instance, mGluR5 is expressed in the Merkel epithelial receptor cells [76], and in an isolated rat sinus hair follicle preparation a non-competitive mGluR1 antagonist produced profound and long-lasting depression of mechanically evoked firing of Merkel cell endings [34]. These findings highlight the role of glutamate neurotransmission in peripheral transduction of sensory stimuli and its involvement in the modulation of the earliest step of sensory information processing [44].

#### 2.2.2. Group II

mGluR2/3 immunolabeling was found in the soma and in the peripheral and central terminals of DRG neurons [77]. Approximately 40% to 52% of DRG cells showed mGluR2/3-IR, which was present almost exclusively in small cells and was coincident with mGluR8-IR in 30% of cells [35,77]. More than 75% of these cells were also labeled with IB4 and their central terminals distributed preferentially in lamina III, with weaker staining in laminae III and IV. This immunoreactivity was almost abolished after dorsal rhizotomy, providing further support for the presence of mGluR2/3 in primary afferent terminals. These receptors may act modulating presynaptically the primary afferent input, particularly nociceptive input, by negatively regulating glutamate release [71,77].

#### 2.2.3. Group III

It is known that mGluR4, mGluR7 and mGluR8 are widely expressed in nearly all nervous tissues involved in nociception, suggesting their involvement in the molecular basis of pain [69]. Indeed, immunostaining of mGluR4a was detected in DRG neurons, particularly in small- to medium-sized ones, and in a dense patch of terminal-IR in lamina II of the DH [78]. The receptor mGluR7 has been detected by IHC in neurons of DRG and TG [79]. mGluR7 is specifically expressed in peptidergic and large DRG neurons in the rat, in which it is often co-expressed with VR1, and is only rarely found in IB4-positive DRG neurons [80]. It seems that due to its inhibitory nature and presynaptic location, mGluR7 could negatively modulate glutamate release, possibly acting as an auto-receptor [71,79,80]. The receptor is transported to the central and peripheral axon terminals, since the IR for mGluR7 in the superficial layers of the DH is reduced after rhizotomy [79], and it builds up at the proximal site after sciatic nerve ligation [80]. Using IHC and stereological techniques, it has been shown that mGluR8 is expressed by 75% of DRG cells of different sizes, having the highest expression among the three groups of metabotropic receptors (with less prevalence in small neurons). Moreover, there was a substantial fraction of double-labeled cells expressing both mGluR2/3 and mGluR8 [77]. mGluR8 seems to be exclusively located on the presynaptic sites of glutamatergic, GABAergic and monoaminergic terminals, regulating negatively or positively (depending of the cell type) neurotransmitter release [70]. The prevalence of groups II and III in DRG neurons indicate that glutamate could have a substantial inhibitory effect on primary afferent function, reducing and/or modulating sensory input before transmission to the spinal cord [71,77].

In sensory ganglia, most studies of mGluRs have been done on DRGs. Only recently the focus was also directed to the TG. Boye Larsen et al. [81] investigated mGluR expression in the TG of adult Wistar rats, and found mGluR1-IR in small- and medium-sized neurons of the TG, opening a possible participation of the receptor in nociceptive transmission. In addition, these authors reported the expression of mGluR2/3 and mGluR8 in medium- to large-sized neurons.

### 2.3. Glutamate Transporters

#### 2.3.1. Vesicular Glutamate Transporters

Three vesicular glutamate transporters (VGLUTs) are known: Brain-specific Na^+^-dependent inorganic phosphate transporter, VGLUT1, expressed only in a subpopulation of glutamatergic neurons [82,83]; differentiation-associated Na^+^-dependent inorganic phosphate transporter, VGLUT2, highly homologous to VGLUT1,expressed only in neurons and enriched in subcortical regions [82,83]; and H^+^-dependent glutamate transporter, VGLUT3, structurally and functionally very similar to the other two [83].

The first study that analyzed the mRNA of VGLUTs in DRGs cells identified VGLUT1 in many neurons (and all the large ones), but failed to detect the other two transporters [84]. More recently, however, mRNA for the three transporters was reported, with the following distribution: approximately 45% of large to medium size cells expressed VGLUT1, 69% of cells of all sizes expressed VGLUT2, and 17% of small- and medium-sized cell expressed VGLUT3 [84]. Immunolabeling of the protein VGLUT1 was observed in 12–13% of mostly large- and medium-sized DRG neurons, while a large proportion of DRG neurons (65–90%), mostly of small- and medium-size, were positive for VGLUT2 [31]. VGLUT1 and VGLUT2-IR was also detected in axon terminals in the DH of the spinal cord, with a different pattern of distribution: VGLUT1 was observed in the deep part of lamina II, and laminae III and IV, whereas VGLUT2 was located in lamina I and the superficial part of lamina II [83,84]. Few fibers were IR for VGLUT3 in the DH [84,85]. VGLUT1 and VGLUT2-IR were coexpressed in SP-containing and IB4-binding fibers, indicating that these transporters were present in nociceptive primary afferents, with a VGLUT2 predominance in unmyelinated fibers [82]. These findings, however, were not corroborated in a later study, which failed to find IR for VGLUT1 and VGLUT2 in various peptide-containing axon terminals (CGRP, SP) [86]. Transection experiments were not conclusive, since dorsal root transection reportedly led to a very marked decrease in VGLUT1 immunopositive fibers, leaving unaltered the expression of VGLUT2 [84], whilst in another study axotomy of the sciatic nerve moderately decreased VGLUT3 mRNA expression and did not affect VGLUT1 and VGLUT2 [84,85].

Glutamate transporters were also identified in the TG: mRNA of the three subtypes was detected by qrtPCR, and IR for VGLUT1 and/or VGLUT2 was found in the 80% of TG neurons [87]. Many of the cells co-expressed both VGLUT1 and VGLUT2, and, depending on the study, it is VGLUT1 [87] or VGLUT2 which predominates [83]. This coincidence of VGLUT-IR was also detected in many axon terminals in the superficial layers of the DH [83]. VGLUT3-IR was also detected in many cells of TG and was coincident with VGLUT1 expression in most cases [87].

It should be noted that the three vesicular transporters are also variably expressed in the peripheral branches of the primary sensory neurons. In particular, these transporters have been identified in dermal and epidermal nerves of the glabrous skin, in the piloneural complex in hairy skin, and in a zone of the rat whisker follicle that is densely populated by Merkel cells [31,62].

#### 2.3.2. Excitatory Amino Acid Transporters (EAATs)

EAATs are responsible for the re-uptake of glutamate into cells and it is well established that under normal conditions, the CNS strongly depends on such re-uptake. Of the five EAATs known, EAAT1 (also known GLAST) and EAAT2 (GLT1) are expressed in the membrane of glial cells; EAAT3 (EAAC1) and EAAT4 are present in neurons in the CNS; and EAAT5 is exclusively located in the retina. Several studies have reported that alterations in the expression or activity of EAATs occurs in CNS disorders, including the central mechanisms for the development and maintenance of neuropathic pain. Chronic constriction nerve injury (CCI) in rats elicits changes in the expression and uptake activity of spinal EAATs that contributed to neuropathic pain [88].

Concerning the PNS, little is known about the EAATs distribution in normal conditions, or following peripheral injuries or pathologies. However, it is known that EAAT1 and -3 are present in the DRG and the sciatic nerve with a differential cellular distribution: in DRG, EAAT1 and EAAT3 are located in neurons and satellite glial cells, and EAAT2 is restricted to SGCs, while in the sciatic nerve all three transporters are present in Schwann cells [89].

To our knowledge, it has not been examined whether these transporters play a role in sensory input-dependent plasticity.

## 3. Glutamate Receptors and Transporters in Non-Neuronal Cells in DRG and TG

**Figure 2:**
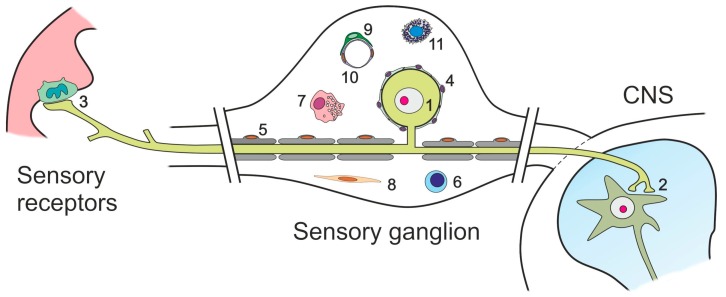
Diagram showing the cellular components of sensory ganglia: (1) cell body of a primary sensory neuron; (2) synaptic boutons from the central projection of the sensory neuron, contacting a second-order neuron in the spinal cord or the trigeminal nuclei of the brain stem; (3) terminal ending of a peripheral branch of the sensory neuron, contacting a skin receptor (Merkel) cell; (4) satellite glial cells, tightly enclosing the cell bodies of sensory neurons; (5) Schwann cells and myelin sheaths; (6) resident T-lymphocyte; (7) resident macrophage; (8) endoneurial and perineurial fibroblast-like cells; (9) pericyte; (10) capillary endothelial cells; and (11) mast cell. Modified after Avendaño, 2010 [90].

In addition to sensory neurons, primary sensory ganglia contain a variety of glial and non-glial cells (Figure 2).

Two types of glial cells abound in sensory ganglia, myelinating and non-myelinating Schwann cells (SC), and satellite glial cells (SGCs), which tightly shroud the somata of primary sensory neurons, and participate in maintaining the homeostasis of the microenvironment, neuronal metabolism and glutamate recycling [91,92,93]. Other cell types include vascular and connective tissue elements, and members of the immune system. Several of these cells have been shown to express glutamate receptors and/or transporters, and therefore are possible targets of glutamate regulation subsequent to alterations in sensory input (Table 1).

### 3.1. Satellite Glial Cells

These neural crest-derived cells are significantly involved in the regulation of glutamate neurotransmission. Glutamate enters the SGCs and is converted into glutamine by glutamine synthetase, which is released and enters the neurons, where it is again transformed into glutamate [92]. Moreover, SGCs are able to release glutamate in response to an increase in intracellular Ca^2+^ concentration, as shown by fluorimetric assays using acutely isolated TG neurons with adhering SGCs [93]. Mechanosensory stimuli or nerve stimulation produce an increase in intracellular Ca^2+^ that spreads not only to neighboring SGCs but also to neighboring neurons, suggesting a possible involvement of SGCs in the processing and transmission of afferent information [26,91].

All three types of ionotropic glutamate receptors and some of the mGLURs have been observed in SGCs, mainly by IHC. GluA4 immunolabeling produced a dense staining of SGCs, whereas little or no staining was detected for subunits GluA2/3 and no staining at all was detected for GluA1 [54]. GluN1, GluN2A and GluN2B were also immunodetected in SGCs of the rat DRG [26,91]. NMDARs present in the SGCs have been involved in inflammatory sensitization of nociceptors, by a proposed mechanism that involves retrograde signaling of glutamate released by spinal cord neurons that activates DRG NMDARs that are located in the SGCs [19]. There is also evidence that NMDARs, in particular those containing GluN2B, may participate in a transglial transmission phenomenon occurring at the DRG in triads formed by neuron-SGC-neuron. If one of these neurons is stimulated with a train of impulses, there is a delayed response in the other neuron, in a so-called “sandwich synapse” that involves Ca^2+^-gated release of ATP in the first synapse (neuron-SGC). This ATP acts on P2Y2 receptors located in the SGC, which releases glutamate that in turn activates GluN2B-containing NMDARs in the second neuron (second synapse, SGC-to-neuron) [101].

Carlton and Hargett [35] first demonstrated the presence of mGluRs in SGCs in the PNS, with the mGluR8 subunit highly expressed and only minimal expression of mGluR1 and mGluR2/3. In agreement with these findings, mGluR1 and mGluR8—but not mGluR2/3—immunoreactivity was also found in the SGCs of the TG of adult rats [18]. In addition, the KA receptor GluK2 is expressed in SGCs [26]. Using Ca^2+^ imaging and specific agonists and/or antagonists, it was proven that these GluRs in the SGCs are functional, further supporting their role in regulating glutamate transmission and neuronal excitability [26,91].

Besides glutamate receptors, SGCs express the glutamate transporters EAAT1, EAAT2 and EAAT3 [89]. The role of GluRs and transporters in SGCs in the excitability of the ganglion neurons, sensitization of primary afferent and pain needs more investigation. Growing evidence highlights the importance of intraganglionic glutamate system in peripheral neuropathies, and SGCs should be taken into account for understanding the mechanisms of glutamatergic signaling, not only under physiological conditions, but also for designing new therapies aimed at dealing with PNS disorders.

### 3.2. Schwann Cells

Cultured SCs express GluN1 and GluN2B, peripheral nerve crush upregulates their expression, and the survival of SCs becomes compromised when the gene encoding GluN1 (Grin1) is silenced, all of which points to a significant role of NMDARs for these cells [102]. In addition, ionotropic receptors have been described in these cells, pointing to an increase of SC signaling after peripheral nerve injury [95].

Recent studies have shown AMPARs mRNA expression in cultured SCs [94], and functional AMPARs expressed in developing SCs [103]. However, when SCs mature and become myelinating, the AMPARs-mediated currents disappear [103].

**Figure 3:**
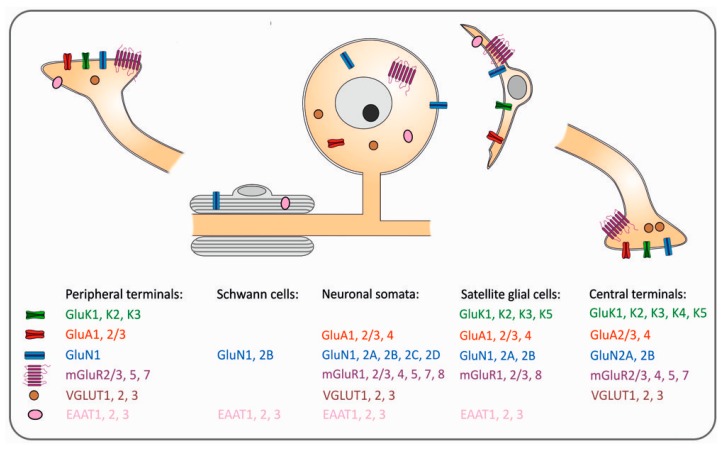
Summary sketch showing the presence of glutamate-related receptors and transporters in the main cell types within the sensory ganglia and the peripheral and central neuronal branches of primary sensory neurons.

In CNS axons, activity-dependent glutamate-mediated signaling from axon to myelin has been demonstrated [104]. Similar mechanisms have not been found in the PNS. However, membrane-bound glutamate transporters EAAT1, EAAT2 and EAAT3 are expressed in the Schwann cells [89], while dependent plasticity is unsolved (Figure 3).

### 3.3. T-Cells, Macrophages and Dendritic Cells

Presence of glutamate-related molecules in peripheral tissues has supported the idea of the role of this amino acid in the function of non-neuronal cells and in neuro-immune relationships. Rat lymphocytes express mRNA coding for GluN1 and Group III mGluRs [105]; T-cells express AMPARs and NMDARs [106,107]; and antigen-presenting cells such as dendritic cells and macrophages express glutamate transporters [108,109].

The expression of these transporters and receptors is modified with the activation of these cells [107,108,110]. Recently, it has been reported that T cells in Multiple Sclerosis patients display elevated levels of GluA3 during relapse and with active disease [111], and that macrophages that do not normally express EAATs do so under inflammatory conditions [110]. In DRG or peripheral nerves, where T-cell and macrophages are present [112,113], no studies have examined whether GluRs are expressed by these cells, and certainly it is unknown whether they play a role in glutamate-related plasticity.

Nevertheless, it is reasonable to speculate that GluRs may be involved in signaling between the neural and the immune systems in PNS. Considering the growing amount of evidence that highlights the role of glutamate in many peripheral neuropathies, many of them with an important inflammatory component, further research is needed to understand the role of glutamate and its receptors and transporters in T-cell and macrophages of sensory ganglia and peripheral nerves.

### 3.4. Fibroblasts

GluN1 is present in the plasma membrane of rat dermal fibroblasts, and it has also been shown, in DRG neuron/fibroblast cocultures, that activation of DRG neurons reduces gap-junctional communication between fibroblasts, which supports the idea that neural activation influences fibroblast networks [114]. However, there is no data about the implication of fibroblast glutamate in sensory transmission or input-dependent plasticity.

### 3.5. Pericytes, Endothelial Cells, Smooth Muscle Cells, and Mast Cells

It is well established that glutamate-induced neuronal activity releases second messengers from neurons or glial cells, thus inducing functional hyperemia. Vasomotor responses from activation of trigeminal fibers are mediated by CGRP, a peptide that plays a key role in the oligemia associated with migraines [115]. Pericytes are known to react to neuronal activity by inducing capillary dilatation when they respond with relaxation to neuron or glial glutamate release [116,117]. Moreover, this glutamate-mediated signaling can be propagated through gap junctions between pericytes or pericytes and endothelial cells. Dermal microvascular endothelial cells, in turn, express mGluR1, mGluR4, and mGluR5. The application of mGluRs agonists increases transendothelial flux, highlighting the role of glutamate in regulating endothelial barrier function [118]. Nevertheless, to our knowledge, no study has examined the glutamate-related neurovascular changes secondary to injury or pathology, or their role in sensory transmission and plasticity.

Mast cells are abundant in DRG and TG but no data exist on their involvement in input-dependent plasticity.

## 4. Glutamatergic Receptors and Transporters in Sensory Ganglia Mobilize in Response to Damage of Peripheral Tissues and Sensory Nerves

The ubiquitous presence of glutamate neurotransmission-related receptors and transporters in small- and medium-sized primary afferent neurons and fibers makes those molecules natural players in the establishment and maintenance of altered noxious processing and chronic pain. When peripheral nerves are injured, robust changes in gene expression and a variety of proteins and other molecules are detected in DRG neurons. These changes appear in trophic factors, neuropeptides, receptors, ion channels, signal transduction molecules and synaptic vesicle proteins among others [22,119]. Regarding glutamate neurotransmission, peripheral nerve axotomy brings about changes in GluRs that persist days or weeks after lesion: mGluR4 and -7 are down-regulated, as well as mGluR7 mRNA, whilst the AMPA subunits GluA3 and -4 increase their expression [80,119,120]. It is interesting to note that the down-regulations observed affect metabotropic receptors that inhibit or decrease neuronal excitability, whereas the up-regulations involve ionotropic receptors mediating an enhancement of neurotransmitter release and/or neuronal excitability. Overall, these changes may lead to potentiation of primary afferent input, which is considered to play a significant role in the development of pain sensitization and perpetuation [121].

After early reports indicated that the subcutaneous administration of ionotropic receptor agonists elicited allodynia and hyperalgesia, which were attenuated by appropriate antagonists [122], the involvement of glutamatergic signaling in nociception and pain would be evidenced at all levels of the peripheral sensory pathway. The stimulation of nociceptors by subcutaneous injections of capsaicin in the rat hind paw caused a pronounced increase in local glutamate release detected by HPLC, which was reduced by the concomitant injection of ionotropic receptor and metabotropic Group I receptor antagonists [123]. In addition, the mechanical allodynia and hyperalgesia that result from inflammation-related activation of AMPA and KA receptors in glabrous skin of the rat hind paw was reduced by the specific deletion of the GluA1—but not GluA2—subunit in nociceptors [44]. In the sensory ganglia, signs of altered glutamatergic neurotransmission have been repeatedly found in several pain models based on nerve injury and/or inflammation. For example, CCI of the sciatic nerve or the infraorbital nerve of rats produced a significant increase in glutamate immunostaining and a concomitant increase in the transporter VGLUT2 in DRG, which points to an augmented release of glutamate due to injury [26]. Likewise, masseter inflammation caused by intramuscular CFA (complete Freund’s adjuvant) injections in male rats produced an up-regulation of mGluR5 and a down-regulation of GluN1 in TG [53].

The central terminal branches of primary afferents make up a critical arena where pain signaling is regulated through modulation of neurotransmitter release. In particular, presynaptic NMDARs are known to facilitate release of glutamate, SP and BDNF from small (C-type) primary nociceptive fibers [21,44]. These receptors in the afferent fiber terminal can play a role as NMDA auto-receptors and lead to the potentiated release of signaling molecules and, eventually, a state of hyperexcitability. Moreover, postsynaptic GluK2 may act synergistically with NMDARs to induce spinal LTP, whereas blockade of NMDARs prevents the primary hyperalgesia induced by C-fiber LTP induction in the superficial spinal DH [124].

Apart from the key role of different types of GluRs in LTP/LTD-induced spinal cord plasticity, and in the development or maintenance of pain, peripheral inflammatory hyperalgesia also depends on glutamate released in DRG leading to an activation of NMDARs in SGCs, which supports a more global role of DRG as the first modulatory center for certain pain conditions [19]. In addition, deletion of VGLUT2 in the whole DRG or just in DRG nociceptors avoids most effects induced by pain triggered by noxious stimuli, peripheral nerve injury or inflammatory pain. If deletion targets VGLUT3 in C-low threshold DRG mechanoreceptors, mechanical hypersensitivity is abolished. These data show that DRG VGLUT2 and VGLUT3 are important players in the transmission of several pain modalities [125].

In sum, a growing body of data supports considering the peripheral glutamatergic system as a promising therapeutic target for pain states, avoiding the risk of interfering with the central glutamatergic processes. In particular, therapeutic interventions on peripheral NMDARs have succeeded in alleviating inflammatory or neuropathic pain conditions [126,127].

## 5. The Glutamatergic System Reacts to Innocuous Manipulation of the Input: Beyond Lesion-Driven and Pain-Related Effects to Experience-Dependent Plasticity in the Brain

During postnatal development, experience-dependent plasticity strongly depends on the activity-dependent maturation of glutamatergic synapses concurrent with the establishment of initial patterns of synaptic connections, and then on the refinement of these connections in adaptation to sensory input [128]. Postsynaptic AMPA and NMDA receptors are critically involved in these processes, initially by the insertion of AMPARs containing Ca^2+^-permeable subunits into “silent” synapses and a change in the subunit composition of NMDARs, and then by a gradual replacement of GluA4 by the Ca^2+^-impermeable GluA2 subunit in AMPA receptors [129,130]. This trafficking is required for developmental synaptic plasticity, including sensory input-dependent dendritic growth, and its blockade prevents normal functional and structural development [5,131,132,133,134]. Past the critical period, dendrites and spines retain a lifelong capacity to change. Growth and retraction of terminal axonal arbors, appearance and disappearance of axonal varicosities and dendritic spines persist, although decaying with time, on a dominant background of connectional stability in animals living under standard housing conditions [135,136,137]. Such changes are boosted in response to prominent and lasting modifications in the signals coming from the periphery, even when such modifications do not derive from any tissue damage, but from innocuous and more “natural” sources of input loss or gain. In adult rodents, these effects have been typically achieved by selectively reducing the active (haptic) touch from some or all whiskers, and by exposing the animals to sensory enriched environments. Both procedures are known to induce a variety of structural and functional changes in cortical and subcortical structures [133,138,139,140,141,142,143,144,145,146,147,148].

Ionotropic glutamate receptors are major players in experience-dependent adult cortical plasticity. AMPARs, in particular those including GluA1, play a role in enabling experience-dependent and long-term depression in granular and supragranular layers of deprived barrels in the barrel cortex in adult mice, since no such depression is observed in GluA1 knock-out mice [10,149]. Likewise, the expression of GluA1-ct-GFP, a construct that blocks the synaptic delivery of GluA1 during potentiation prevents experience-driven plasticity [150]. It was only recently that GluA1 was shown to be a necessary—although not sufficient—factor for the experience-dependent potentiation observed in single spared whisker preparations in adult mice [151]. In contrast, GluA2 and GluA3, two subunits that make the AMPAR less permeable to Ca^2+^, and which are involved in the insertion and stabilization of AMPARs independently of activity, do not differ from controls in layer IV of the barrel cortex several weeks after whisker trimming [152]. Postsynaptic NMDARs are also needed for whisker pairing plasticity in the barrel cortex (produced by unilateral trimming of all whiskers, but for two adjacent ones), which is aborted by the application of an NMDA antagonist [16]. Different types of sensory deprivation in adult rats, including whisker trimming, lead to a reappearance of “silent” synapses in the barrel cortex with a re-expression of the GluN2B subunit, which suggests a reactivation of the conspicuous synaptic plasticity characteristic of postnatal developmental periods [131].

Extended exposure to sensory-enriched environments is known to enhance exploratory behaviors in adult rats, and to bring about a variety of structural and functional changes in the barrel cortex, such as expansion of neuronal somata and dendrites, expansion of cortical thickness, increase in number and size of synapses and so on [144,153,154,155,156]. Even short exposures to enrichment induce detectable up-regulation of genes (mostly immediate-response genes) in the barrel cortex [156,157], but changes in expression of genes related with synaptic transmission and neuronal structure only occurred after longer periods of enrichment [158]. At the protein level, GluR expression was found to be regulated in different forebrain regions by long exposures to enrichment. For example, GluA1 and GluN2B and -2A significantly increased in whole forebrain homogenates of mice after long-term exposure to enrichment, and these changes correlated with improvements in behavioral performance on several memory tasks [159]. In the rat hippocampus, in particular, an increase in GluN2A was observed after enrichment, together with a decrease in an excitatory amino acid carrier transporter (EAAT3), which could be related to an increase of glutamate in the synapse [155].

Compared to the glutamatergic mechanisms in dendrites and spines, the participation of presynaptic structures in activity- and experience-dependent changes in primary sensory cortex has been less well studied. Sensory deprivation decreases glutamate release probability at the granular to supragranular synapses in the barrel cortex [160,161]. However, it is unclear whether increased sensory stimulation enhances glutamate release. It has indeed been proposed that postsynaptic potentiation (as in LTP) may come with a reduced presynaptic release probability, as a homeostatic adjustment to the increased input [162,163]. Activation of presynaptic NMDA (and cannabinoid) receptors results in synaptic depression [164] especially in young animals. This effect is diminished by normal sensory experience, but it may be reinstalled in adulthood by sensory deprivation [18,33]. The subunit composition of these presynaptic receptors is variable, depending on the brain region and the developmental stage of the subject. In adults, the 2B subunit predominates in the cortex, and is also present in the spinal cord and brain stem in combination with subunits 2C and 2D [33,156]. Metabotropic receptors abound in presynaptic locations, both inside and outside active synaptic zones. These receptors mainly include Group II and Group III types, but some studies have also identified mGluR1 and -5 in a fraction of synaptic terminals [165]. While substantial advances have been made in recent years on the roles played by these receptors in the regulation of neurotransmission [161,166], their involvement in experience-dependent plasticity is practically unknown.

## 6. The Glutamatergic System and Experience-Dependent Plasticity in the Sensory Ganglia

As reviewed in the above sections, the DRG and the TG are rich in glutamate-related receptors and transporters, as are the primary sensory neurons and other cells that these ganglia contain (Table 1). For a long time, mature primary sensory neurons were considered relatively passive relays for transferring sensory information to central nervous structures. The massive cellular and molecular changes in these neurons that followed axotomy or other nerve injuries [22,119,167,168,169] were considered to represent adaptive, self-protective and regenerative responses of the damaged cell. Even when chronic pain conditions developed after nerve or peripheral tissue injury, the primary sensory neuron was easily dismissed as a merely ancillary or subordinate actor [170]. Not surprisingly, sensory ganglia have been practically overlooked in experience-dependent plasticity studies.

Recent studies by our group [96,171], however, showed that the terminal afferents of primary sensory neurons, and the expression of many glutamate-related genes in the adult TG of rats change after chronic exposure to an enriched environment, or repeated unilateral whisker trimming. While the explanation of those molecular changes was complicated by the existence of lateral asymmetries in a good number of genes, some conclusions emerged from this study: (1) Environmental enrichment led to a reduction of metabotropic receptors-encoding genes on the right side, which suggests a facilitation of synaptic transmission at least on the right side. (2) The gene encoding the subunit 2B of the NMDA receptor decreases on the right side and does not change on the left, but the protein significantly increases on both sides after enrichment. This finding parallels the increased contents in this subunit in the forebrain after long-term exposure to enrichment [159]. (3) The AMPA receptor subunits GluA1 and GluA4 are reduced on both sides following enrichment, which could be interpreted to favor synaptic facilitation [98]. (4) Unilateral haptic deprivation by repeated whisker trimming on the right side gives rise to bilateral modifications in several glutamate-related genes and receptors; overall, these changes consisted in an extensive upregulation of gene expression in the right TG, and a more limited downregulation in the left TG. (5) Increases in gene expression on the right side after deprivation mainly involved the genes encoding GluA1, GluA2, GluN2B and mGluR3, with variable changes in protein content. (6) Gria4, the gene coding for GluA4, was bilaterally upregulated in TG after unilateral deprivation, with significant increases of the subunit protein only on the right TG. (7) GluN2B significantly increased in the right TG and did not vary in the left, where its encoding gene Grin2B was markedly down-regulated.

Some of these gene and protein changes paralleled effects reported after peripheral nerve lesions or chronic pain conditions [38,96,120], and even some of the long-term molecular changes observed in CNS after deprivation or enrichment (see Section 5). However, our results just scratch the surface of really understanding the mechanisms whereby sensory ganglia react and adapt to the changing conditions imposed by an altered sensory input. It goes without saying that: (1) sensory ganglia are complex structures that lodge a variety of cells—not just neurons—some of which utilize glutamate receptors or transporters; and (2) only cell bodies and a minor part of their neurites are located in the ganglia, thus whatever gene or protein change is detected in the ganglia might be due to phenomena taking place far away from them.

## 7. Concluding Note

It seems reasonable to speculate that the numerous molecular changes in sensory ganglia that result from manipulations of the sensory input should be at least instrumental to determine the functional and structural changes that appear further up in the sensory pathway. To what extent these relationships may be causal is unexplored, and probably difficult to demonstrate. However, it may be affirmed that the molecular, functional and structural plasticity of primary sensory neurons and their accompanying cells lay a fundamental ground for adaptation strategies, learning processes and recovery from lesions. Not a minor issue, only by better understanding how these early stations of sensory processing react to input distortions could new and more effective chronic pain therapies be developed.

## Figures and Tables

**Table 1:** Summary of the glutamate-related genes and proteins that are expressed in DRG and/or TG neurons and glial cells.

Gene Protein	Neuron			Satellite Glial Cell	Schwann Cell	Whole Ganglia
	Body	Central Projection	Peripheral Projection			
**Grik1** **Gluk1**	+[46,47]	+[47] +[42]	+[58,94]	+[94]	−[95] *	+[44]
**Grik2** **Gluk2**	+[47] −[26]	+[47] +[42]	+[58,94]	+[26,94]	+[95] *	+[44]
**Grik3** **Gluk3**	+[47]	+[47] +[42]	+[58,94]	+[94]	+[95] *	+[44]
**Grik4** **Gluk4**	+[47]	+[51]			+[95] *	+[44]
**Grik5** **Gluk5**	+[47]	+[51]			+[95]*	+[44]
**Gria1** **GluA1**	+[46,55]		+[58]	−[54]	+[95] *	+[96]
**Gria2/3** **GluA2/3**	+[38,46,54,55]	+[36]	+[94]	+ [94]	+[95] *	+[96] +[96]
**Gria4** **GluA4**	+[38,54]	+[26,36]		+[26,54]	+[95] *	+[96]
**Grin1** **GluN1**	+[37,46] +[37,38]		+[58,94]	+[91,94]	+[95] * +[95] *	+[96] +[37]
**Grin2A** **GluN2A**	+[26,37]	−[97]		+[26]	+[95] *	+[37,96] −[37]
**Grin2B** **GluN2B**	+[37]	+[97]		+[91]	+[95] * +[95] *	+[37,96] +[37,96]
**Grin2C** **GluN2C**	+[37]				+[95] *	+[37,98] +[37]
**Grin2D** **GluN2D**	+[37]				+[95] *	+[37,98] +[37]
**Grin3A** **GluN3A**					+[95] *	
**Grin3B** **GluN3B**					+[95] *	
**Grid1** **GluD1**						
**Grid2** **GluD2**						
**Grm1** **mGluR1**	+[35,81]			+[81]		+[98]
**Grm2/3** **mGluR2/3**	+[35,77,81]	+[77]	+[77]	−[81]		+[96] +[96]
**Grm4** **mGluR4**	+[99]	+[72]				+[96]
**Grm5** **mGluR5**	+[72]	+[72]	+[75]			+[96]
**Grm6** **mGluR6**						
**Grm7** **mGluR7**	+[74,77]	+[74,77]				+[96]
**Grm8** **mGluR8**	+[26,35,81]			+[26,35,81]		+[96]
**Slc1a3** **EAAT1**	+[89]		+[89]	+[89]	+[89]	+[89]
**Slc1a2** **EAAT2**	−[89]		+[89]	+[89]	+[89]	+[89]
**Slc1a1** **EAAT3**	+[89]		+[89]	−[89]	+[89]	+[89]
**Slc1a6** **EAAT4**						
**Slc1a7** **EAAT5**						
**Slc17a7** **VGLUT1**	+[85] +[87,100]	+[84,100]	+[100]			+[85,87] +[84]
**Slc17a6** **VGLUT2**	+[85] +[87,100]	+[84,100]	+[100]			+[85,87]
**Slc17a8** **VGLUT3**	+[85] +[87]	+[84]	+[31]			+[85,87] +[87]

* Data derived only from cultured cells.
